# Supplementary material for: A Likelihood Approach for Real-Time Calibration of Stochastic Compartmental Epidemic Models
Source: PLoS Comput Biol. 2017 Jan 17;13(1):e1005257. doi: 10.1371/journal.pcbi.1005257 (PMC5240920; doi:10.1371/journal.pcbi.1005257)
Supplement: S1 File — (TAR.GZ) [file pcbi.1005257.s014.tar.gz › HSPH_Online-SI-Revision/IPoi09/n1-extreme/n1-extreme_BENCH_table-8.pdf]

1	R0	{2.95019, 14.2854}	{1.95081, 12.2686}	{{1.14983, 6.31117}, {2.87797, 17.9732}}	{33.958, 26.5262}	inf	3.	1.93	{0., 5.}	77.	0.69457	newly	0.1	1.72	{0., 4.}	1724.	0.663595	Reff	2.93839	1.94508	{1.14868, 2.87509}	33.8996	ar	924.	346.03	{1., 917.}	62.7825
2	R0	{2.67565, 16.946}	{2.07951, 14.897}	{{1.55644, 9.52864}, {2.46533, 19.391}}	{22.5253, 19.3682}	inf	30.	19.5667	{2., 43.}	61.3333	0.54487	newly	15.	11.6833	{1., 25.}	67.4444	0.577019	Reff	2.55525	2.0478	{1.56566, 2.39938}	20.2237	ar	926.	721.867	{10., 889.}	22.1094
3	R0	{2.40066, 16.6543}	{2.09209, 12.2417}	{{1.29927, 4.25564}, {2.76849, 18.2127}}	{19.8402, 30.1214}	inf	14.	9.88333	{0., 23.}	64.6429	0.580205	newly	8.	9.43333	{0., 20.}	105.833	0.620951	Reff	2.34544	2.10905	{1.40845, 2.70205}	17.3901	ar	916.	642.8	{2., 907.}	30.0328
4	R0	{1.9294, 15.4361}	{1.48715, 11.7138}	{{1.06501, 5.62019}, {1.86902, 17.4454}}	{25.4962, 30.751}	inf	10.	5.26087	{0., 12.}	70.4348	0.779866	newly	5.	3.6087	{0., 8.}	87.8261	0.808764	Reff	1.89467	1.50582	{1.11415, 1.96782}	23.4876	ar	796.	330.565	{2., 784.}	59.5095
5	R0	{2.92859, 19.5393}	{1.4536, 16.2739}	{{1.28197, 11.0915}, {1.59945, 19.8325}}	{50.3651, 17.0814}	inf	28.	19.1765	{2., 50.}	58.8235	0.480341	newly	16.	9.61765	{0., 25.}	67.0956	0.677473	Reff	2.7968	1.38548	{1.16587, 1.57546}	50.4621	ar	927.	498.412	{56., 669.}	46.2339
6	R0	{1.79622, 12.3148}	{2.21905, 14.1659}	{{1.77826, 9.06807}, {2.7811, 19.8588}}	{26.0984, 28.4018}	inf	35.	50.6429	{5., 105.}	89.6939	0.370205	newly	31.	29.8036	{4., 67.}	61.4631	0.40389	Reff	1.6651	2.09391	{1.65529, 2.52622}	27.5583	ar	734.	844.929	{730., 921.}	16.1395
7	R0	{2.45855, 17.1776}	{2.12635, 13.1012}	{{1.34668, 7.40677}, {2.7943, 18.4042}}	{21.4853, 27.2499}	inf	24.	10.6364	{1., 24.}	72.3485	0.775627	newly	13.	8.04545	{0., 22.}	81.7016	0.778886	Reff	2.38971	2.18473	{1.44786, 2.75965}	17.9862	ar	871.	626.303	{3., 920.}	31.4546
8	R0	{2.20009, 15.519}	{2.05955, 14.3806}	{{1.67732, 9.44073}, {2.43839, 19.391}}	{12.6374, 22.3973}	inf	26.	109.825	{19., 190.}	336.203	0.627044	newly	18.	55.7937	{18., 86.}	222.31	0.528679	Reff	2.00208	1.51168	{0.988894, 2.07687}	26.5909	ar	839.	805.492	{681., 891.}	7.50516
9	R0	{2.06493, 9.6414}	{2.129, 4.77845}	{{1.27121, 2.19254}, {2.85474, 7.40677}}	{23.877, 54.8368}	inf	4.	32.71	{0., 99.}	814.75	1.23295	newly	3.	88.51	{0., 326.}	2947.67	1.46269	Reff	2.04222	1.97078	{1.2674, 2.67585}	22.7038	ar	777.	406.28	{1., 920.}	56.9035
10	R0	{2.60775, 19.6936}	{1.64193, 14.7692}	{{1.34829, 9.41429}, {1.95665, 19.2013}}	{37.0366, 25.079}	inf	29.	29.5778	{2., 71.}	69.8851	0.479793	newly	16.	16.0667	{1., 38.}	65.9722	0.48758	Reff	2.47997	1.55044	{1.28993, 1.84512}	37.4817	ar	889.	575.756	{36., 778.}	35.2356
11	R0	{1.99168, 8.13555}	{1.98562, 12.1649}	{{1.2743, 5.40945}, {2.85038, 18.33}}	{25.1357, 62.0227}	inf	4.	8.37681	{0., 24.}	170.29	0.587251	newly	3.	7.68116	{0., 23.}	232.367	0.721037	Reff	1.97375	2.07657	{1.28323, 2.84753}	26.2893	ar	772.	603.841	{2., 932.}	38.4077
12	R0	{1.79304, 9.18941}	{1.95081, 12.2686}	{{1.14983, 6.31117}, {2.87797, 17.9732}}	{31.4583, 46.5518}	inf	3.	1.93	{0., 5.}	77.	0.69457	newly	4.	1.72	{0., 4.}	82.5	0.992014	Reff	1.78587	1.94508	{1.14868, 2.87509}	31.4176	ar	725.	346.03	{1., 917.}	65.7669
13	R0	{1.84016, 18.3431}	{1.94978, 16.3079}	{{1.59546, 12.272}, {2.2194, 19.5128}}	{10.9102, 14.3893}	inf	36.	58.7736	{8., 110.}	96.8029	0.362259	newly	12.	28.5283	{4., 53.}	163.208	0.435355	Reff	1.71319	1.70221	{1.3929, 1.98187}	12.5525	ar	762.	735.113	{604., 856.}	13.7647
14	R0	{1.94221, 9.85971}	{2.40638, 13.0555}	{{1.83586, 8.17412}, {2.86656, 17.1903}}	{28.4183, 42.8466}	inf	16.	44.0156	{5., 89.}	195.41	0.456739	newly	13.	35.5	{4., 68.}	197.596	0.472672	Reff	1.86646	2.22923	{1.59317, 2.75407}	26.7208	ar	805.	855.984	{734., 935.}	13.0804
15	R0	{1.78578, 12.0541}	{1.85834, 15.5602}	{{1.56965, 10.7905}, {2.11476, 19.2116}}	{9.54764, 32.3317}	inf	16.	37.2727	{5., 68.}	156.818	0.420765	newly	14.	19.9091	{3., 37.}	77.2727	0.320324	Reff	1.68578	1.70421	{1.40547, 1.99121}	10.8906	ar	732.	726.364	{598., 826.}	11.5698
16	R0	{1.94463, 15.6503}	{1.84477, 14.1415}	{{1.17434, 8.35044}, {2.53075, 19.3211}}	{23.3109, 22.1031}	inf	8.	9.59211	{0., 27.}	117.928	0.806803	newly	4.	6.31579	{0., 16.}	159.211	0.819403	Reff	1.92518	1.83604	{1.17631, 2.4997}	22.3787	ar	757.	483.618	{1., 901.}	47.4327
17	R0	{2.08901, 16.4314}	{1.64761, 16.6424}	{{1.29096, 9.52864}, {1.99753, 19.5128}}	{22.0783, 19.842}	inf	17.	10.3091	{1., 27.}	61.6043	0.544175	newly	8.	5.89091	{0., 12.}	66.3636	0.609675	Reff	2.03887	1.6877	{1.31045, 2.01002}	18.0394	ar	826.	573.982	{21., 818.}	30.7572
18	R0	{2.76419, 18.56}	{2.07344, 10.1708}	{{1.25474, 3.26445}, {2.75598, 15.6124}}	{26.0467, 45.3143}	inf	14.	32.2557	{0., 98.}	202.026	0.789774	newly	7.	29.7463	{0., 97.}	386.354	0.867086	Reff	2.70338	2.02745	{1.30295, 2.612}	25.6376	ar	920.	643.507	{2., 919.}	30.3618
19	R0	{2.3821, 17.1399}	{2.00387, 10.7854}	{{1.1315, 4.47101}, {2.74988, 18.2815}}	{23.3182, 39.9721}	inf	12.	21.55	{0., 55.}	197.417	1.20176	newly	10.	29.85	{0., 80.}	320.5	1.28412	Reff	2.34637	1.89654	{1.1097, 2.62397}	24.6905	ar	883.	407.33	{1., 921.}	55.6886
20	R0	{2.78314, 19.6127}	{2.16866, 16.7586}	{{1.81389, 11.7348}, {2.42205, 19.5287}}	{22.0786, 14.6363}	inf	70.	58.6393	{12., 106.}	50.7026	0.374779	newly	32.	29.2295	{6., 59.}	53.6373	0.384294	Reff	2.44916	1.902	{1.56357, 2.1878}	22.3407	ar	943.	800.574	{769., 882.}	15.1035
21	R0	{1.85124, 14.6794}	{2.11254, 14.2695}	{{1.67732, 9.44073}, {2.52892, 19.3027}}	{18.4608, 19.2614}	inf	18.	22.2167	{3., 54.}	86.5741	0.435368	newly	15.	14.6	{1., 30.}	82.6667	0.533263	Reff	1.78274	2.06396	{1.63542, 2.47897}	19.6729	ar	730.	733.233	{16., 902.}	22.9817
22	R0	{2.1028, 14.9852}	{2.03328, 11.8497}	{{1.25656, 4.21286}, {2.68803, 17.7287}}	{20.788, 29.8027}	inf	26.	43.5167	{0., 98.}	127.756	0.625855	newly	17.	29.	{0., 65.}	122.745	0.585251	Reff	2.01659	1.93053	{1.21781, 2.44629}	18.7833	ar	840.	672.983	{10., 916.}	25.7996
23	R0	{1.86601, 10.2157}	{1.8352, 13.5183}	{{1.11523, 7.7153}, {2.53741, 19.3027}}	{24.4238, 42.8368}	inf	8.	7.28169	{0., 20.}	93.4859	0.705748	newly	6.	4.60563	{0., 14.}	95.0704	0.802978	Reff	1.84548	1.80563	{1.12157, 2.48974}	23.2157	ar	747.	484.746	{1., 896.}	47.2048
24	R0	{2.34032, 10.0992}	{1.95081, 12.2686}	{{1.14983, 6.31117}, {2.87797, 17.9732}}	{26.9894, 38.7686}	inf	2.	1.93	{0., 5.}	90.5	0.606525	newly	1.	1.72	{0., 4.}	176.	0.703595	Reff	2.3333	1.94508	{1.14868, 2.87509}	26.9549	ar	887.	346.03	{1., 917.}	62.2492
25	R0	{1.91548, 15.3401}	{1.71174, 13.5813}	{{1.13227, 6.31117}, {2.26107, 19.0665}}	{21.1715, 23.8463}	inf	10.	13.0189	{0., 30.}	104.906	0.624646	newly	8.	8.18868	{0., 19.}	87.7358	0.655331	Reff	1.88292	1.73486	{1.20629, 2.2475}	18.068	ar	765.	519.321	{3., 854.}	38.5177
26	R0	{2.75283, 10.6721}	{1.92583, 12.9514}	{{1.15964, 8.35044}, {2.688, 18.33}}	{30.8575, 35.2206}	inf	2.	4.61972	{1., 10.}	169.014	0.450015	newly	1.	3.46479	{0., 7.}	291.549	0.584899	Reff	2.73907	1.93509	{1.2015, 2.67032}	30.1906	ar	926.	492.155	{1., 920.}	47.101
27	R0	{2.08367, 19.7849}	{1.4634, 7.36382}	{{1.21159, 3.78882}, {1.86094, 12.8742}}	{30.4227, 62.7806}	inf	51.	34.	{7., 63.}	49.8599	0.40895	newly	27.	31.9762	{6., 62.}	70.4586	0.355827	Reff	1.89822	1.29799	{0.815957, 1.89538}	33.8527	ar	833.	539.786	{285., 804.}	35.8172
28	R0	{2.64334, 17.1765}	{2.32673, 9.82663}	{{1.5451, 4.64686}, {2.91762, 13.5542}}	{16.6409, 43.1169}	inf	29.	27.2698	{3., 62.}	62.7805	0.46293	newly	16.	29.5238	{2., 65.}	123.611	0.485125	Reff	2.5191	2.29839	{1.60304, 2.80161}	14.8383	ar	934.	807.698	{595., 942.}	13.8829
29	R0	{2.87008, 13.3872}	{1.59843, 13.8842}	{{1.19306, 6.88118}, {1.95859, 19.1609}}	{44.3071, 26.5025}	inf	6.	14.0682	{2., 32.}	170.833	0.433577	newly	4.	7.68182	{1., 20.}	150.	0.457808	Reff	2.82129	1.61531	{1.22879, 2.03659}	42.7455	ar	941.	549.773	{18., 826.}	41.5757
30	R0	{2.06105, 19.6275}	{2.21989, 16.8122}	{{2.01705, 13.609}, {2.42625, 19.4475}}	{9.60008, 14.3752}	inf	103.	151.939	{64., 214.}	65.4016	0.248716	newly	49.	66.9848	{31., 91.}	52.35	0.203427	Reff	1.55609	1.30719	{0.773227, 1.93088}	27.1811	ar	747.	845.182	{795., 892.}	13.1638
31	R0	{2.30764, 13.435}	{1.92583, 12.9514}	{{1.15964, 8.35044}, {2.688, 18.33}}	{23.513, 25.4626}	inf	14.	17.5775	{0., 44.}	121.73	0.853682	newly	11.	13.2113	{0., 31.}	126.633	0.9387	Reff	2.26841	1.87861	{1.18819, 2.53484}	22.354	ar	877.	492.155	{1., 920.}	45.7674
32	R0	{2.30565, 11.9237}	{2.24191, 9.59967}	{{1.43579, 4.47101}, {2.80569, 14.1724}}	{17.3805, 28.2418}	inf	8.	9.12698	{1., 23.}	87.1032	0.483914	newly	11.	11.254	{0., 27.}	94.9495	0.65061	Reff	2.26876	2.29783	{1.57287, 2.82441}	15.9549	ar	853.	721.952	{3., 911.}	21.489
33	R0	{2.24031, 18.582}	{1.26329, 15.1181}	{{1.04117, 10.1673}, {1.54363, 18.9003}}	{43.611, 19.8313}	inf	23.	6.26316	{0., 20.}	73.913	0.948395	newly	10.	3.18421	{0., 10.}	74.4737	0.991492	Reff	2.17982	1.26544	{1.01787, 1.57479}	41.9476	ar	864.	227.789	{3., 582.}	73.6355
34	R0	{2.33306, 15.9759}	{2.26418, 13.73}	{{1.67042, 9.06807}, {2.86656, 17.7758}}	{16.223, 19.7072}	inf	16.	12.8438	{1., 31.}	73.6328	0.563569	newly	12.	9.42188	{1., 22.}	78.5156	0.63221	Reff	2.27473	2.27098	{1.83009, 2.81122}	14.3668	ar	854.	717.469	{6., 932.}	22.4898
35	R0	{2.48357, 18.3573}	{1.56336, 14.4438}	{{1.05515, 8.34284}, {2.44675, 18.834}}	{38.7102, 22.8624}	inf	17.	5.89041	{0., 19.}	82.917	1.07072	newly	9.	3.43836	{0., 11.}	88.5845	1.07137	Reff	2.43887	1.57306	{1.06559, 2.41609}	36.8711	ar	835.	346.466	{1., 880.}	60.6529
36	R0	{2.59548, 19.9046}	{1.83922, 12.7643}	{{1.32705, 6.77341}, {2.30108, 19.1478}}	{29.1376, 35.8728}	inf	18.	19.3137	{2., 38.}	68.0828	0.401415	newly	13.	13.0196	{1., 29.}	69.2308	0.475672	Reff	2.50983	1.80141	{1.33725, 2.20657}	28.2257	ar	863.	678.078	{349., 860.}	21.6322
37	R0	{2.29641, 16.9868}	{1.55919, 13.9796}	{{1.32705, 8.42022}, {1.8189, 19.2972}}	{32.1032, 23.921}	inf	21.	27.814	{6., 55.}	80.7309	0.361265	newly	10.	15.1163	{2., 35.}	93.0233	0.396611	Reff	2.18159	1.49814	{1.24029, 1.74512}	31.3281	ar	877.	602.558	{389., 742.}	31.2933
38	R0	{2.65743, 15.4569}	{2.17624, 14.7532}	{{1.75469, 10.0119}, {2.53519, 19.255}}	{18.522, 17.1886}	inf	29.	43.7937	{6., 92.}	99.9453	0.40914	newly	16.	25.3968	{4., 54.}	102.579	0.41794	Reff	2.52987	2.01091	{1.61785, 2.38697}	20.576	ar	897.	790.317	{689., 908.}	12.4418
39	R0	{2.67953, 14.5132}	{1.56336, 14.4438}	{{1.05515, 8.34284}, {2.44675, 18.834}}	{42.2039, 22.0216}	inf	2.	9.24658	{0., 24.}	441.781	0.867187	newly	1.	5.45205	{0., 15.}	530.137	0.813431	Reff	2.67149	1.55749	{1.06559, 2.25655}	41.7867	ar	894.	346.466	{1., 880.}	61.5672
40	R0	{1.94594, 11.928}	{2.3202, 12.7845}	{{1.75445, 8.39294}, {2.83341, 16.8013}}	{24.4962, 26.4053}	inf	31.	52.3065	{5., 116.}	106.087	0.398223	newly	31.	36.9194	{7., 73.}	67.794	0.358156	Reff	1.84475	2.17127	{1.64208, 2.59959}	24.1583	ar	796.	837.823	{734., 937.}	14.5465
41	R0	{1.66297, 10.0047}	{1.34059, 5.23362}	{{1.25656, 3.82585}, {1.40191, 6.88118}}	{19.6037, 48.5742}	inf	25.	20.963	{2., 43.}	58.5185	0.44935	newly	18.	27.5185	{2., 61.}	96.5021	0.441735	Reff	1.51995	1.04088	{0.701327, 1.38088}	32.8249	ar	716.	430.074	{145., 567.}	40.089
42	R0	{2.91568, 16.4453}	{1.95081, 12.2686}	{{1.14983, 6.31117}, {2.87797, 17.9732}}	{33.3262, 29.1606}	inf	9.	3.66	{0., 10.}	77.3333	1.03953	newly	8.	3.7	{0., 9.}	91.75	1.14153	Reff	2.88652	1.93724	{1.14868, 2.87509}	33.1821	ar	919.	346.03	{1., 917.}	62.7282
43	R0	{2.55898, 15.8889}	{1.84477, 14.1415}	{{1.17434, 8.35044}, {2.53075, 19.3211}}	{29.2468, 22.0578}	inf	3.	3.59211	{0., 9.}	92.5439	0.485981	newly	3.	2.60526	{0., 7.}	85.0877	0.671895	Reff	2.54619	1.86413	{1.18463, 2.51237}	27.985	ar	904.	483.618	{1., 901.}	46.8634
44	R0	{2.28649, 15.9017}	{1.83317, 13.893}	{{1.27357, 8.09734}, {2.35866, 19.2013}}	{21.8426, 23.023}	inf	13.	9.	{1., 24.}	71.4286	0.607581	newly	10.	5.48214	{0., 16.}	71.9643	0.691879	Reff	2.24076	1.84496	{1.29914, 2.40441}	20.5017	ar	880.	526.196	{4., 867.}	40.4363
45	R0	{2.65121, 18.6339}	{1.95837, 11.5287}	{{1.15409, 5.21967}, {2.83341, 18.4504}}	{28.4952, 38.8725}	inf	4.	2.05	{0., 5.}	78.25	0.779692	newly	1.	2.61	{0., 4.}	261.	0.71782	Reff	2.6353	1.95139	{1.15063, 2.83058}	28.3923	ar	928.	335.55	{1., 907.}	63.8847
46	R0	{2.52291, 18.0251}	{1.33071, 13.2976}	{{1.03756, 7.48339}, {1.77361, 18.9248}}	{47.2549, 28.1252}	inf	13.	5.1087	{0., 18.}	72.7425	0.9586	newly	3.	3.02174	{0., 10.}	103.623	0.760275	Reff	2.4775	1.39419	{1.0553, 1.94177}	43.7258	ar	894.	262.174	{2., 769.}	70.6741
47	R0	{2.22618, 17.1225}	{2.24278, 13.132}	{{1.44485, 5.65591}, {2.78517, 18.33}}	{17.8934, 26.7053}	inf	9.	10.9531	{1., 29.}	84.2014	0.452615	newly	3.	8.51563	{1., 23.}	226.563	0.54468	Reff	2.18388	2.2921	{1.77334, 2.75965}	16.5538	ar	850.	718.156	{7., 931.}	21.8824
48	R0	{2.04405, 16.073}	{2.24165, 8.66629}	{{1.44762, 4.01966}, {2.82285, 12.6436}}	{22.6862, 46.0817}	inf	18.	67.8596	{9., 155.}	295.906	0.575517	newly	12.	69.614	{13., 144.}	491.813	0.720477	Reff	1.96229	2.08061	{1.41416, 2.6118}	19.7422	ar	779.	829.246	{711., 935.}	17.2151
49	R0	{2.84396, 18.097}	{1.95837, 11.5287}	{{1.15409, 5.21967}, {2.83341, 18.4504}}	{31.6345, 37.7519}	inf	14.	5.29	{0., 15.}	91.6429	1.27072	newly	11.	7.19	{0., 20.}	119.182	1.37248	Reff	2.79846	1.93769	{1.14717, 2.79502}	31.4313	ar	952.	335.55	{1., 907.}	64.7532
50	R0	{1.85341, 18.0975}	{2.17033, 6.73119}	{{1.4031, 2.60601}, {2.84073, 10.1535}}	{27.3816, 62.806}	inf	61.	136.034	{36., 251.}	140.644	0.404255	newly	26.	104.431	{43., 165.}	306.167	0.601237	Reff	1.67178	0.739822	{0.427827, 1.11413}	56.3732	ar	754.	811.517	{695., 934.}	16.249
